# Discovery of Notch Pathway-Related Genes for Predicting Prognosis and Tumor Microenvironment Status in Bladder Cancer

**DOI:** 10.3389/fgene.2022.928778

**Published:** 2022-06-30

**Authors:** Xianchao Sun, Shiyong Xin, Weiyi Li, Ying Zhang, Lin Ye

**Affiliations:** ^1^ Department of Urology, Shanghai East Hospital, School of Medicine, Tongji University, Shanghai, China; ^2^ Department of Urology, The Second Affiliated Hospital of Anhui Medical University, Hefei, China

**Keywords:** notch pathway, bladder cancer, prognostic, immune microenvironment, risk

## Abstract

**Background:** Notch signaling is a key regulator of immune cell differentiation and linked to autoimmune diseases, tumorigenesis and tumor-induced immunomodulation. An abnormally activated Notch signaling pathway contributes to almost all of the key features of cancer, including tumor angiogenesis, stemness, and epithelial-mesenchymal transition. Consequently, we investigated Notch pathway-related genes for developing prognostic marker and assessing immune status in bladder cancer.

**Methods:** The Cancer Genome Atlas (TCGA) and Gene Expression Omnibus (GEO) databases were utilized to analyze RNA-seq data for bladder cancer. Cluster subtypes were identified using the NMF algorithm. In order to establish a prognostic risk signature, the least absolute shrinkage and selection operator (Lasso) and Cox regression analysis was utilized. GSEA was carried out to investigate the molecular mechanisms. Immune cell infiltration levels in bladder cancer were calculated using the CIBERSORT algorithm. External clinical tissue samples were used to validate the expression levels of signature genes.

**Results:** Based on the NMF algorithm, bladder cancer samples were divided into two cluster subtypes and displayed different survival outcome and immune microenvironment. A six-gene risk signature (DTX3L, CNTN1, ENO1, GATA3, MAGEA1, and SORBS2) was independent for prognosis and showed good stability. The infiltration of immune cells and clinical variables were significantly different among the risk groups of patients. Response to immunotherapy also differed between different risk groups. Furthermore, the mRNA expression levels of the signature genes were verified in tissue samples by qRT-PCR.

**Conclusion:** We established a 6-gene signature associated with Notch pathway in bladder cancer to effectively predict prognosis and reflect immune microenvironment status.

## Introduction

The Notch gene was discovered in *Drosophila* in 1917 and was named “Notch” by geneticist Morgan, because its partial deletion caused a dentate notch on the wing margin of the fly (Andersson et al., 2011). In subsequent studies, it was found that the Notch genes were widely present in various organisms, ranging from invertebrates to mammals with high homology (Kunze et al., 2020). Gradually, Notch and related genes have been recognized as an evolutionarily highly conserved and play key role as a determinant of cell fate in embryonic development and differentiation, influencing cell proliferation, cell cycle, apoptosis, and serving as a critical role in both physiological and pathological conditions (Capaccione and Pine, 2013). However, the nature of its action depends on the tissue cell context. Notch can exhibit both pro- and anti-cancer effects in different tissue cells, and even in different stages of the same tumor (Trindade and Duarte, 2020). In breast cancer, Notch is an oncogene that promotes tumorigenesis (Bai et al., 2020), whereas it plays the role of oncogenic suppressor in skin tumors (Panelos and Massi, 2009). In non-small cell lung cancer, Notch is a pro-oncogene, while it acts as an oncogene suppressor in small cell lung cancer (Leonetti et al., 2019). During the early stage of cervical cancer, Notch1 is a cancer-promoting factor, but in the advanced stage of cervical cancer, Notch1 is transformed into a cancer-inhibiting factor (Maliekal et al., 2008). The multifaceted nature of Notch signaling suggests the requirement to investigate the mechanism of Notch in various types of tumors.

Bladder cancer (BCa) is one of the most common tumors of the urinary system (Siegel et al., 2022). Clinically, invasive BCa has higher malignancy, early metastasis and higher mortality than non-muscle invasive BCa. Surgical treatment includes radical cystectomy and urinary diversion, both of which are difficult and traumatic (Patel et al., 2020). Patients also have poor postoperative quality of life. Research on BCa should provide valuable insights into early diagnosis, gene-targeted therapy, and a better prognosis.

Existing studies have demonstrated that Notch signaling pathway is not only critical in tumorigenesis and development, but also indispensable in prognosis, and has great promise as a therapeutic target for tumors (Aster et al., 2017). Notch and other signaling mechanisms form a complex network of interactions (Hibdon et al., 2019). Therefore, targeting Notch may provide a new strategy for cancer prevention and therapeutic drug development. Loss of Notch1 gene copy number is present in nearly 50% of BCa, and increased extracellular regulatory protein kinase (ERK) 1/2 phosphorylation due to inactivation of the Notch signaling pathway is a driver of highly invasive uroepithelial carcinogenesis (Rampias et al., 2014). In contrast to Notch1 inactivation, Notch2 expression was upregulated in BCa, and high levels of both EMT and stem cell marker expression were also detected and associated with poor prognosis (Hayashi et al., 2016).

Currently, immune cells in the tumor microenvironment (TME) contribute significantly in the biological function of tumor (Hinshaw and Shevde, 2019). Through continuous exploration and in-depth analysis, there are many new advances in understanding of the complexity of Notch signaling pathway in different tumor immune cells, and the molecular mechanisms of Notch on cell function (Janghorban et al., 2018; Meurette and Mehlen, 2018; Grazioli et al., 2022). Targeting genes related to Notch may have different effects on cancer prevention and treatment.

In the present study, we investigated the impact of Notch signaling pathway-related genes in BCa prognosis and immune microenvironment. Using bioinformatics methods and clinical tissue samples validation, we established a 6-gene signature as a predictor of prognosis and immune infiltration status in BCa.

## Materials and Methods

### Data Collection

Human Notch pathway-related gene sets were downloaded from the Molecular Signature Database (MSigDB) (Liberzon et al., 2015), and 428 genes (Supplementary Table S1) were obtained from seven Notch-related pathways (Supplementary Table S2). BCa patient samples and corresponding clinicopathological information were acquired from TCGA database and GEO database (GSE13507).

### Cluster Subtypes Identification

428 genes from the TCGA dataset were extracted and genes with significant differential expression were selected. BCa samples were clustered using non-negative matrix factorization (NMF) clustering algorithm (Zhuo et al., 2020). With the *R* package “NMF”, we set the number of clusters k from 2 to 10, and determined the average contour width of the matrix.

### Gene Set Enrichment Analysis

Molecular mechanisms were investigated using GSEA (Subramanian et al., 2005). We retrieved the gene sets “c2. cp.kegg.v7.4. symbols” and “c5. go.v7.4. symbols” from MSigDB database. Statistical significance was defined as a *p*-value less than 0.05. Enrichment analyses were conducted using the *R* package “clusterProfiler”.

### Estimation Value of the Risk Signature

Kaplan-Meier analysis was used to compare the clinical survival outcome between the low- and high-risk groups. Various clinicopathological variables were also analyzed for their association with the risk model.

### Comprehensive Analysis of Immune Characteristics

In order to investigate the immune landscape with different cluster groups, we calculated the degree of immune cell infiltration. Common methods to evaluate the immune landscape include TIMER, XCELL, MCPCOUNTER, QUANTISEQ, CIBERSORT-ABS, EPIC, and CIBERSORT (Luan et al., 2021). Correlation coefficients were presented as a heat map. Between low- and high-risk subgroups, BCa samples were examined for their immune profiles by importing their expression data into CIBERSORT and iterating 1000 times to estimate the relative proportions of immune cells (Mo et al., 2020). The results also displayed as a landscape map showing the proportion of immune cells and clinicopathological factors.

### The Ability of the Risk Model to Evaluate Response to Clinical Treatment

Due to the critical role of immune checkpoint molecules in immunotherapy, we analyzed the correlation between the risk model and the expression levels of immune checkpoint molecules and visualized data as a box line diagram. Moreover, the IC50 (half maximal inhibitory concentration) of some important anti-tumor drugs were also calculated in the risk model. Differences in the IC50 were identified by the Wilcoxon signed-rank test and the “pRRophetic” and “ggplot2” tools in the *R* environment (Geeleher et al., 2014).

### Construction of the Nomogram

The nomogram depicts the prognosis of cancer by using a visual model. To predict the prognosis of BCa patients, we constructed a nomogram. The accuracy and consistency of the prognostic model were evaluated using a calibration plot.

### Clinical Patients and Bladder Specimens

Sixty paired normal and tumor tissues were collected from BCa patients who underwent surgery at the Second Affiliated Hospital of Anhui Medical University (Hefei, China). They had diagnostic criteria according to the WHO classification and received no preoperative treatment. Informed consent was obtained from each patient before inclusion in the study, and an ethical approval was obtained from the Ethics Committee of the Second Affiliated Hospital of Anhui Medical University.

### RNA Extraction and qRT-PCR

TRIzol (Invitrogen, United States) was used to extract the total RNA. qRT-PCR was conducted based on the manufacturer’s instruction. β-actin was an internal control. Fold-changes were calculated by the 2^-∆∆Ct^ method. Primer information is shown in Supplementary Table S3.

### Statistical Analysis

Bioinformatic analyses were conducted using *R* version 4.1.1. For comparing continuous data, Student’s t or Wilcoxon test were used. Spearman correlation analysis was used to analyze the correction between the risk signature and immune cells. All statistical *p*-values were two-sided, and *p* < 0.05 was considered statistically significant.

## Results

### Sample Typing Based on Notch Pathway-Related Genes

**FIGURE 1:**
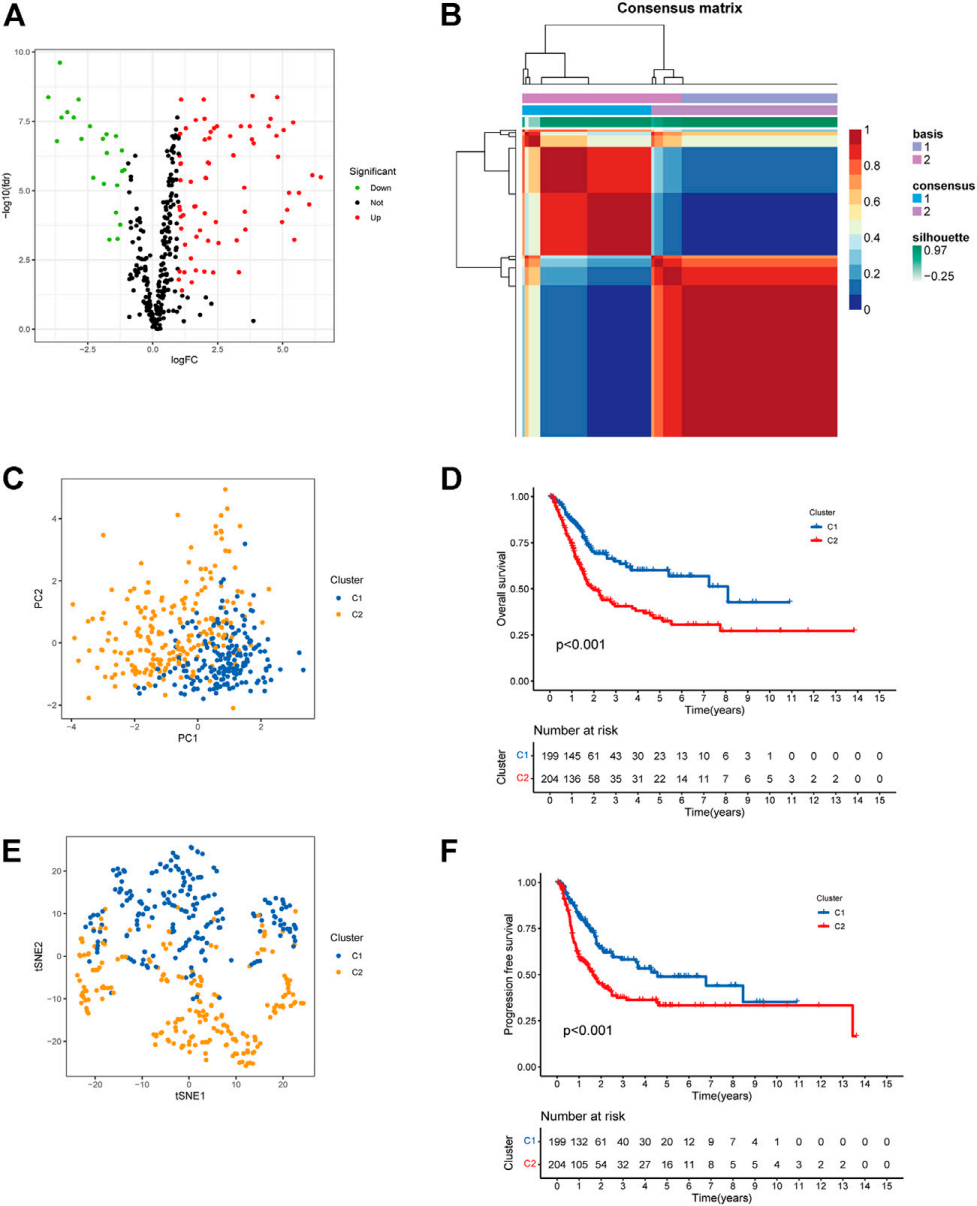
Cluster subtypes were identified. **(A)** Volcano map displayed the differentially expressed Notch pathway-related genes in TCGA-BLCA. Red: up-regulation, blue: down-regulation. **(B)** NMF clustering consensus map. **(C)** PCA plot in the two cluster groups. **(D)** t-SNE analysis in the two cluster groups. **(E)** Overall survival analysis of two clusters. **(F)** Progression-free survival analysis of two clusters.

Seven Notch pathway-related gene sets were downloaded from MSigDB. The related gene expression of BCa was obtained using data from the TCGA bladder cancer cohort (TCGA-BLCA). To identify genes with differential expression, the “limma” *R* package was utilized. The differential expression of 95 Notch pathway-related genes were screened in TCGA-BLCA (*p* < 0.05, Figure 1A, Supplementary Table S4). After that, BCa samples were clustered by the NMF method. Cophenetic, dispersion, and silhouette all indicate that k = 2 is an optimal number of clusters (Figure 1B, Supplementary Figure S1). Two distinct directions of cluster samples distribution were indicated by PCA plots and t-SNE analysis (Figures 1C,D) (Gralinska et al., 2022). Overall survival (OS) and progression-free survival (PFS) prognostic relationships between Cluster 1 (C1) and Cluster 2 (C2) show that subgroup C1 has a better prognosis than subgroup C2 (Figures 1E,F, log rank *p* < 0.001).

### Immune Infiltration in Two Subtypes

**FIGURE 2:**
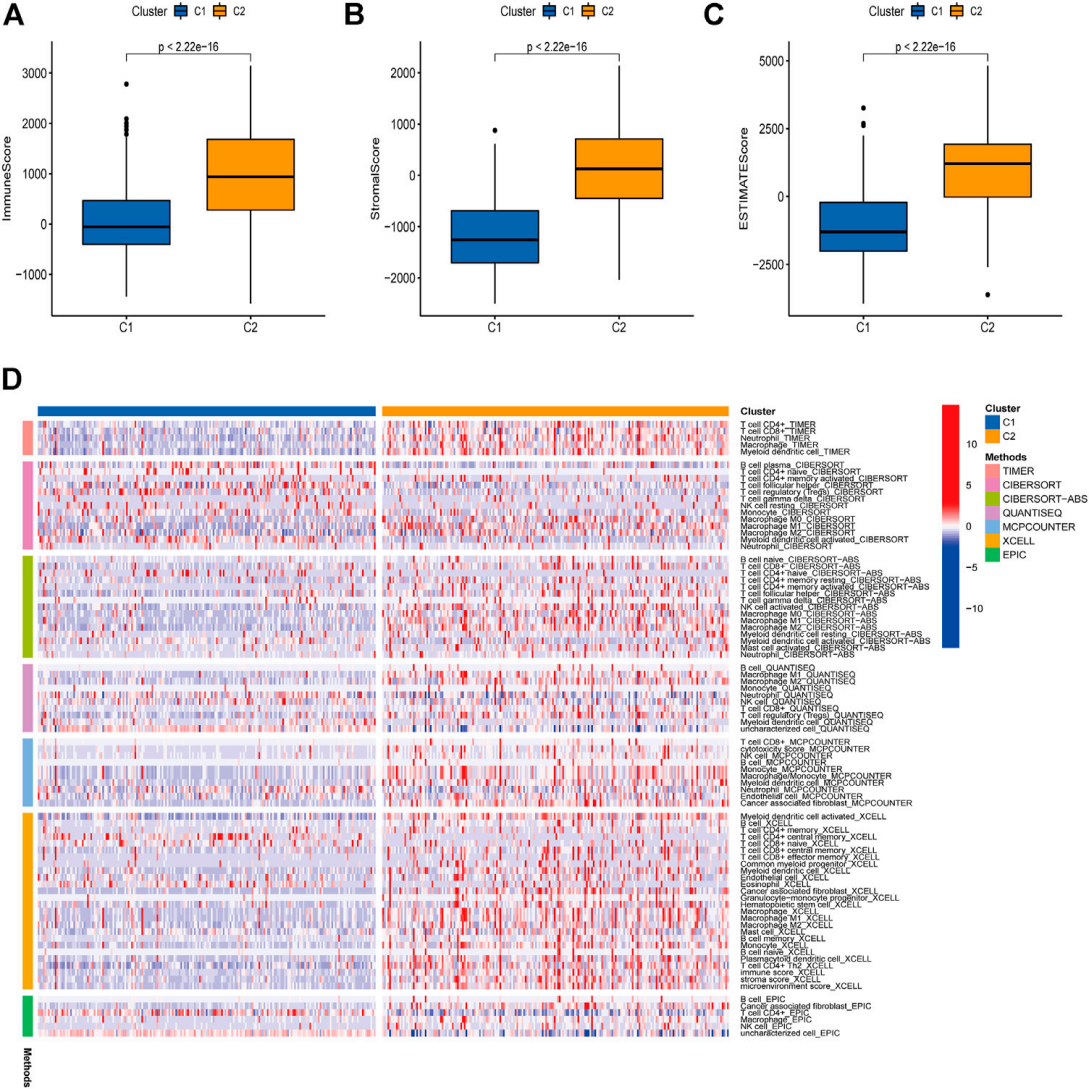
Immune infiltration in two subtypes. A correlation was investigated between the Immune and Stromal scores of the two clusters using the ESTIMATE method **(A–C)**. Immune infiltration cells in the two cluster groups were visualized **(D)**.

We then analyzed the immune infiltration status in the two cluster groups. ESTIMATE algorithm was applied to investigate the correlation between the two clusters in Immune scores and Stromal scores (Ma et al., 2021). The results showed that C2 group showed higher Immune scores, Stromal scores, and Estimate scores than C1 group (Figures 2A–C). We further investigated the immune infiltration cells in C1 and C2 groups, and TIMER, CIBERSORT, CIBERSORT-ABS, QUANTISEQ, MCPCOUNTER, XCELL, and EPIC methods were used to calculate the proportions of different immune cells in different groups. As shown in Figure 2D, immune infiltrating cells were more enriched in the C2 group compare to C1 group, indicating a differential immune cells infiltration status between the two cluster subtypes.

### Construction of Risk Model Associated With Prognosis

We randomly divided BCa samples into a training cohort and a testing cohort in a 7:3 ratio.

**FIGURE 3:**
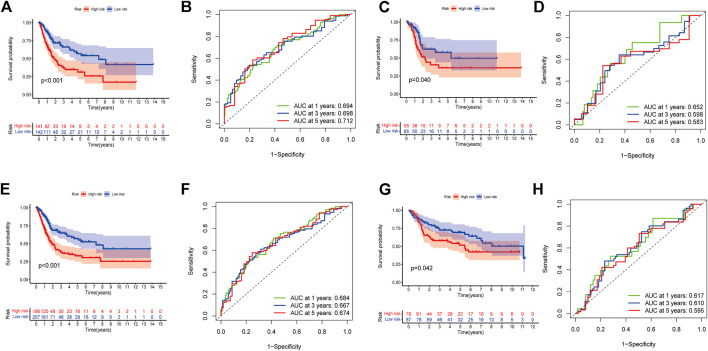
Establishment of a prognostic risk model. **(A)** Group survival curves from the TCGA training cohort. **(B)** ROC curve for the TCGA training cohort. **(C)** Group survival curves from the TCGA testing cohort. **(D)** ROC curve for the TCGA testing cohort. **(E)** Group survival curves from the entire TCGA cohort. **(F)** ROC curve for the entire TCGA cohort. **(G)** Group survival curves from the GSE13507 cohort. **(H)** ROC curve for the GSE13507 cohort.

**FIGURE 4:**
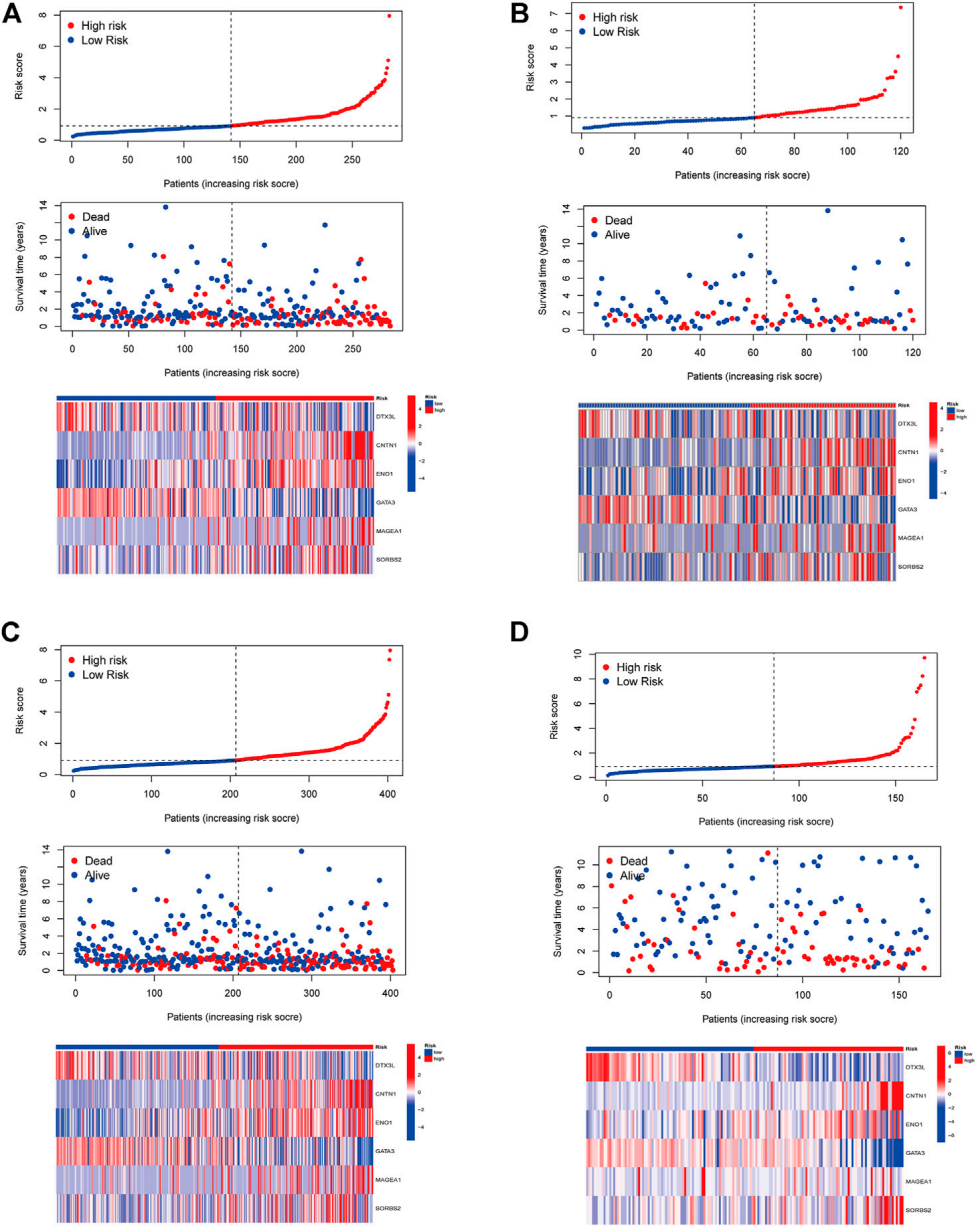
The distribution of the BCa patients with risk scores in different cohorts. Distribution of patients by risk score in low- and high-risk groups and the expression levels of the six signature genes were displayed as a heatmap in TCGA training cohort **(A)**, TCGA testing cohort **(B)**, entire TCGA cohort **(C)**, and GSE13507 cohort **(D)**.

In order to identify prognosis-related genes in the training set, a univariate Cox proportional hazard analysis was performed. A *p*-value threshold of less than 0.05 identified 11 genes associated with significant prognostic differences (Supplementary Table S5). The hub genes were further selected using Lasso regression and multivariate Cox analyses in order to narrow the gene range and construct a highly accurate prognostic model (Supplementary Figure S2). Six target genes were determined by combining the analysis. The 6-gene signature formula was as follows: *Risk score = expression level of DTX3L × (-0.490) + expression level of CNTN1 × (0.230) + expression level of EN O 1 × (0.412) + expression level of GATA3 × (-0.107) + expression level of MAGEA1 × (0.146) + expression level of SORBS2 × (0.284).* Following this, we divided the samples into two risk subgroups based on the risk score, plotted the K-M curve, as shown in Figure 3A, and found significant differences between the two groups (*p* < 0.001). After that, we calculated the area under the curve (AUC) for 1, 3, and 5 years, which were 0.694, 0.698, and 0.712, respectively (Figure 3B). Additionally, we evaluated the model’s robustness by using the same coefficients in other cohorts. Among the testing cohort, the AUC values were 0.652, 0.598, and 0.583, respectively (Figure 3D); among all TCGA cohort, they were 0.684, 0.667, and 0.674, respectively (Figure 3F). The AUC values for the GSE13507 cohort at 1, 3, and 5 years were 0.617, 0.610, and 0.595, respectively (Figure 3H). In both cohorts of patients, similar results were obtained, and there were significant survival differences (Figures 3C,E,G). As the risk elevated, the number of deaths increased, and the number of surviving patients decreased among these cohorts. The expression levels of the six signature genes between low- and high-risk subgroups were displayed as heat map (Figure 4, Supplementary Table S6). Based on these results, the constructed signature of risk has a high level of robustness and could be used to predict BCa patient prognosis across different cohorts.

### Using Risk Model as Independent Prognostic Factor

**FIGURE 5:**
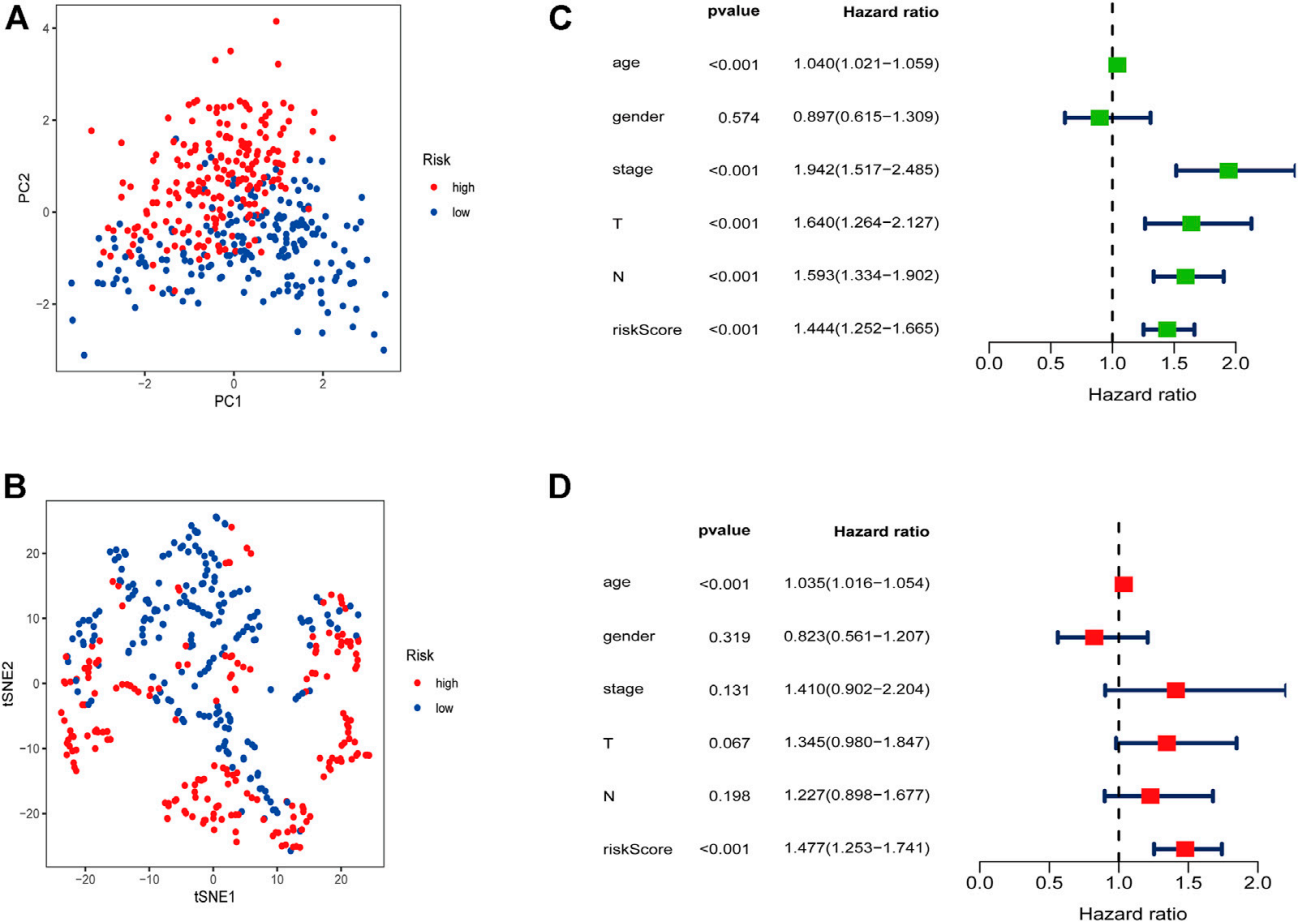
Risk signature was an independent predictor of prognosis. **(A)** PCA plot in the two risk groups. **(B)** t-SNE analysis in the two risk groups. Univariate **(C)** and multivariate **(D)** Cox analysis.

Two distinct directions of risk samples distribution were indicated by PCA plots and t-SNE analysis (Figures 5A,B). We next confirmed the independence of the model. Univariate Cox regression analysis indicated that age, clinical stage, T stage, N stage, and risk score were closely related to survival (Figure 5C). According to multivariate analysis, only age (HR = 1.035, 95% CI = [1.016–1.054], *p* < 0.001) and risk score (HR = 1.477, 95% CI = [1.253–1.741], *p* < 0.001) were significantly related to survival (Figure 5D). These results demonstrated that this 6-gene signature was an independent factor predicting prognosis.

### Association Between the Risk Model With Clinical Characteristics

**FIGURE 6:**
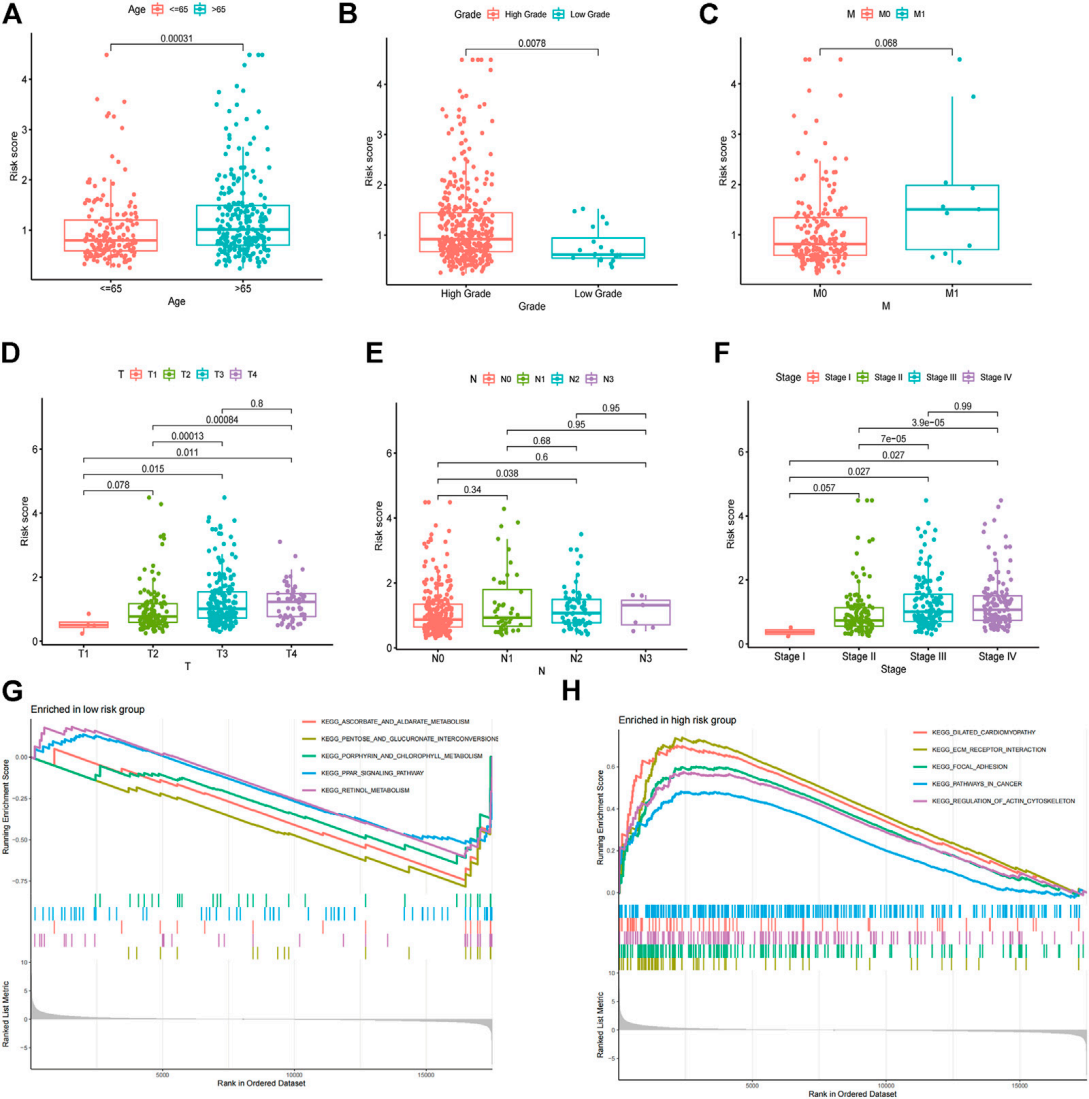
Association between the risk model with clinical characteristics. Correlation analysis of risk score with age **(A)**, grade **(B)**, M stage **(C)**, T stage **(D)**, N stage **(E)**, and clinical stage **(F)**. GSEA enrichment analysis of biological activities between the low **(G)** and high **(H)** risk groups.

The risk score was further compared among patients with different clinical characteristics. There was a statistically significant correlation between the risk score and clinical characteristics such as age, grade, T stage, and clinical stage (Figures 6A–F). Also, there was no significant differences between earlier stages for T stage (T1 vs. T2, *p* = 0.078), clinical stage (Stage I vs. Stage II, *p* = 0.057). The data limitation in the public database TCGA might be the reason for this. Gene sets enriched in specific risk subgroups were identified using GSEA. High-risk sample gene sets were enriched in pathways related to focal adhesion, ECM receptor interaction, and pathways in cancer, while low-risk sample gene sets were enriched in PPAR signaling pathway and metabolism (Figures 6G,H).

### Correlation Between the Risk Model and Immunity

**FIGURE 7:**
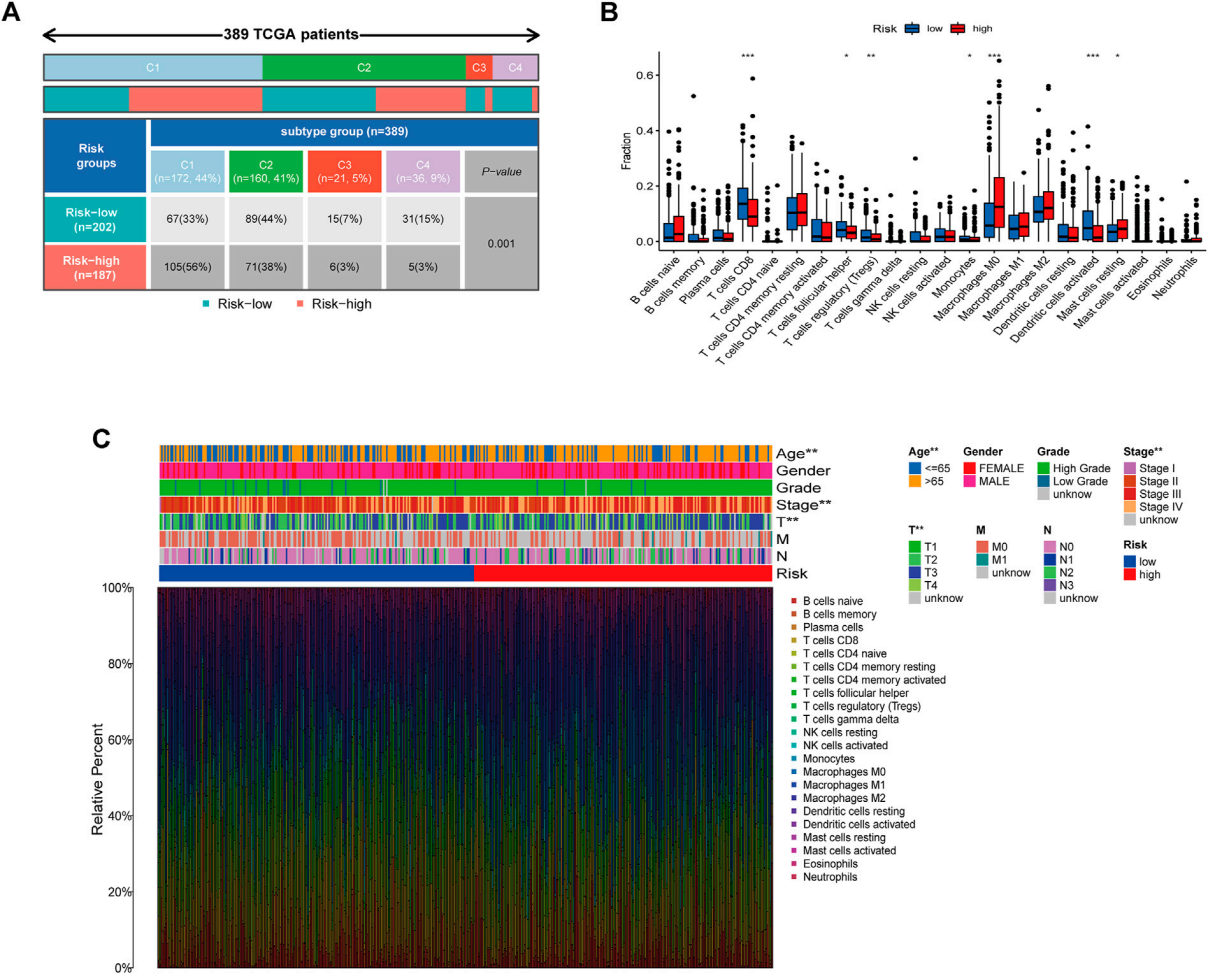
Correlation between the risk model and immunity. **(A)** Different immune subtypes distributed in the two risk groups. **(B)** Two risk subgroups with different proportions of immune cells. **(C)** Clinical features of risk with the immune landscape. *p < 0.05, **p < 0.01,***p < 0.001.

A 33 diverse cancer immune subtype classification has described the immune landscape of BCa according to the immune expression characteristics of four representative signatures: C1 (wound healing), C2 (IFN-γ dominant), C3 (inflammatory), and C4 (lymphocyte depleted) (Thorsson et al., 2018). We therefore found that a higher proportion of C1 was distributed in the high-risk subgroup, while a higher proportion of C4 in low-risk subgroup (*p* = 0.001) (Figure 7A). CIBERSORT was applied to evaluate the relative proportions of 22 kinds of immune cells in the TME to examine the indicative roles of this risk model. A significant correlation was found between high-risk subgroups and M0 macrophages and Mast resting cells, while the low-risk subgroup was significantly associated with CD8 T cells, T follicular helper cells, T regulatory cells (Tregs), Monocytes, and dendritic activated cells (Figure 7B). Other methods to evaluate the immune landscape also provide a significant immunity difference (Supplementary Figure S3). Figure 7C illustrated the relationship between clinical and immune infiltration cells characteristics of different subgroups at risk.

**FIGURE 8:**
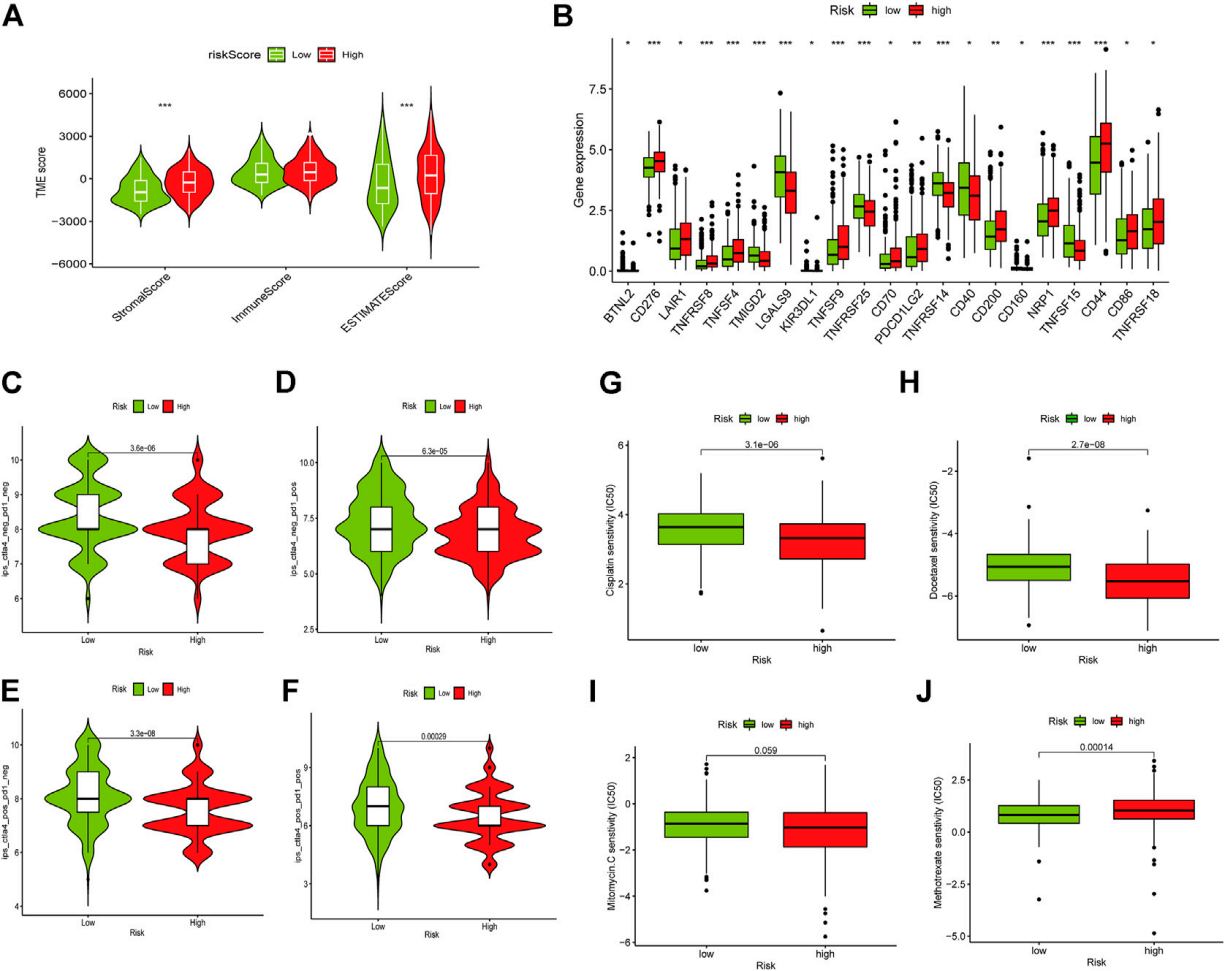
Role of the risk signature in immunotherapeutic responses. **(A)** ESTIMATE algorithm was used to investigate the correlation between the two groups in Immune scores and Stromal scores. Some immune checkpoints were investigated between the two risk groups **(B)**. **(C–F)** The correlation between immunophenoscore and different risk groups. Low risk subgroup was positively correlated with a higher IC50 of Cisplatin **(G)**, Docetaxel **(H)**, and Mitomycin C **(I)**, while a lower IC50 of Methotrexate **(J)**. *p < 0.05, **p < 0.01,***p < 0.001, ns *p* > 0.05.

As well, we investigated the correlation between the two groups in Immune scores and Stromal scores. The results showed that high-risk group showed higher Stromal scores and Estimate scores than low-risk group (Figure 8A). Moreover, we explored the potential of the risk model for predicting the response to immune checkpoint inhibitors (ICIs). Some immune checkpoints were investigated between the two risk groups, and the expression of CD276, TNFSF4, CD70, NRP1, and CD86 were markedly higher in the high-risk subgroup, while the expression of TMIGD2, LGALS9, CD40, and TNFSF15 were significantly higher in the low-risk subgroup (Figure 8B). However, the expression levels of some key immune checkpoints such as PD-1, PD-L1, CTLA4, LAG3, and TIGIT displayed no significant statistical differences between low- and high-risk subgroups (Supplementary Figure S4). It is worth exploring in depth to reveal the underlying correlations. For investigating the capacity of risk predicting response to immunotherapeutic, immunophenogram analysis was undertaken to investigate association between immunophenoscore (IPS) and different risk subgroups (Wu et al., 2021). Findings showed that the low-risk subgroup exhibited higher IPS compared with the high-risk subgroup, which implied that low risk score patients might exhibited higher positive response to immunotherapy (Figures 8C–F). Chemotherapy is an effective strategy for cancer treatment. We further analyzed the correlation between risk score and chemotherapeutic efficacy. We found that low risk subgroup was positively associated with a higher IC50 of Cisplatin, Docetaxel, and Mitomycin C, while a lower IC50 of Methotrexate, indicating a different distribution of targeted IC50 agents in low and high risk subgroups (Figures 8G–J).

### Construction of the Nomogram

**FIGURE 9:**
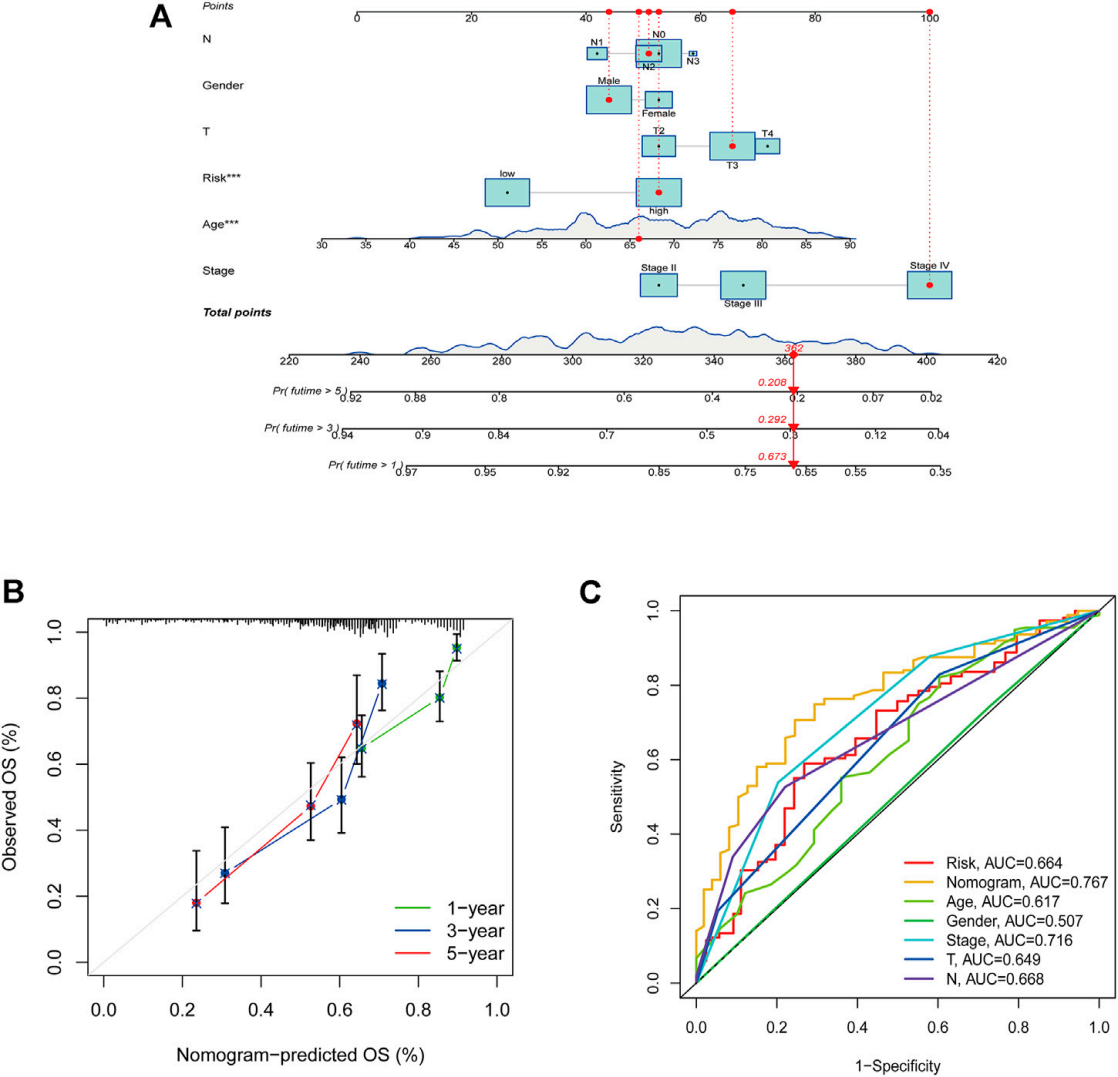
Construction of the nomogram with risk score. **(A)** A nomogram to predict survival. **(B)** A calibration plot for prediction. **(C)** AUC for the nomogram.

By combining the clinicopathological characteristics and risk score, we were able to construct a nomogram that could be clinically applied. Figure 9A illustrated the nomogram with risk score. Calibration plots were shown for 1-, 3-, and 5-year periods to show the performance of the nomogram (Figure 9B). Additionally, the nomogram displayed the highest accuracy in predicting survival (AUC = 0.767) compare to other independent factors (Figure 9C).

### Clinical Validation of This Risk Model

**FIGURE 10:**
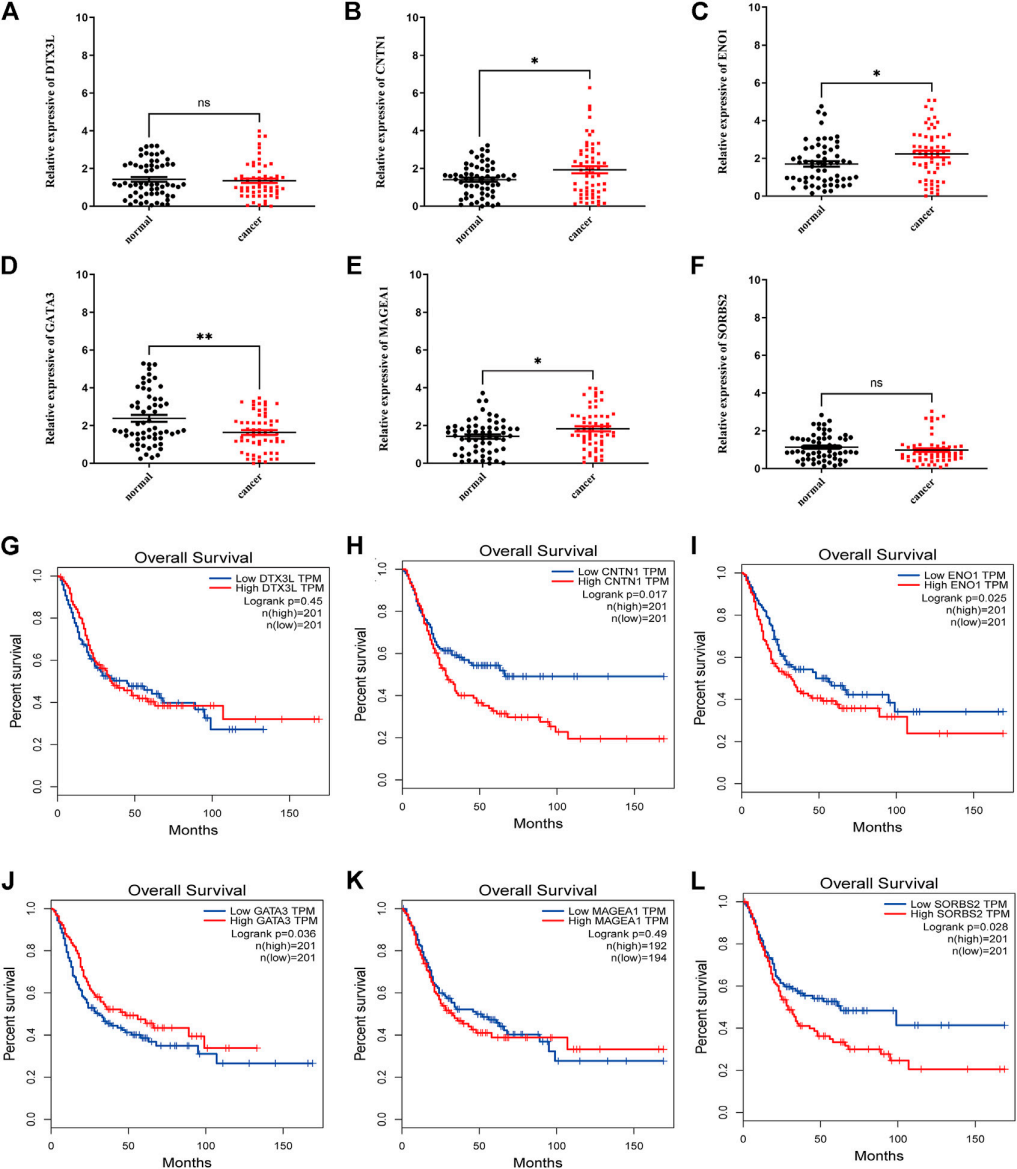
Clinical validation of this risk model genes. qRT-PCR analysis of DTX3L, CNTN1, ENO1, GATA3, MAGEA1, and SORBS2 mRNA levels in tissue samples **(A–F)**. **(G–L)** GEPIA survival analysis of the six genes. *p < 0.05, **p < 0.01,***p < 0.001, ns *p* > 0.05.

In addition to the above results, 60 cases of tissue specimens of BCa were analyzed. We verified the mRNA expression of the six signature genes in cancer and normal tissues by qRT-PCR. The findings also showed that the mRNA expression of CNTN1, ENO1, and MAGEA1 were higher in tumor tissues, whereas the mRNA expression of GATA3 was higher in normal tissues (Figures 10A–F). Gene Expression Profiling Interactive Analysis (GEPIA) database was applied to analyze the associations between the six signature genes and OS in BCa (Tang et al., 2017). High expressions level of CNTN1, ENO1, SORBS2 as well as low expression level of GATA3 were closely correlated with poorer survival outcomes of BCa patients (Figure 10G-L). The Human Protein Atlas (HPA) database was used to show the immunohistochemical staining for signature genes in normal and cancer bladder tissues (Supplementary Figure S5). Our workflow for this study was displayed in Supplementary Figure S6.

## Discussion

Tumor biomarkers are mainly used in clinical practice to detect primary tumors and screen for risk-high groups, contributing to the prognosis and treatment outcome of patients (López-Cortés et al., 2021). In recent years, some BCa-related tumor markers have been used in the clinic to help detect clinically occult cancer, such as bladder tumor antigen and epithelial cells marker (Stefan-van Staden et al., 2020; Gouin et al., 2021). Hence, exploring BCa prognosis-related biomarkers would have good clinical application prospects.

Notch signaling pathway is a highly conserved pathway that regulates cell proliferation, apoptosis and determines cell fate and development. The classical Notch pathway is initiated by the binding of the extracellular structural domain of Notch receptor to a ligand on an adjacent cell. In mammals, there are five ligands (Jagged1, Jagged2, Delta-like1 (DLL1), DLL3 and DLL4), and ligand and receptor cell type-specific and spatial expression can regulate Notch signaling. Notch receptor-ligand interactions are mediated by γ-secretase for secondary cleavage, releasing the intracellular structural domain (NICD), which is translocated to the nucleus to act on transcriptional regulation of target genes (Kopan and Ilagan, 2009). The most important functions of the Notch signaling pathway in the immune system are the differentiation of T and B lymphocyte lineages and T cell activation (Stanley and Guidos, 2009). Notch pathway could affect functional dendritic cell maturation and dendritic cell-mediated T cell responses, while T cells express Notch receptors and ligands expressed on dendritic cells (Cheng et al., 2010). Notch2 plays a major role in IL-19-mediated maturation of lung dendritic cells, which may have potential implications for the involvement of antigen-presenting cells in autoimmune diseases (Hoffman et al., 2011).

TME mainly includes blood vessels, immune cells, fibroblasts and extracellular matrix, plays an important role in the development of tumorigenesis. TME can influence the biological behavior of tumors by regulating tumor cell gene expression, epigenetics, and the interaction of tumor cells with their surrounding environment. Previous studies have shown that Notch pathway mRNA expression is inversely correlated with Treg number and its FOXP3 mRNA expression in tumor tissues, and that downregulation of Notch promotes Treg infiltration and breast cancer phenotype (Ortiz-Martínez et al., 2016). Activation of Notch signaling pathway promotes macrophage polarization to M1 type and increases their anti-tumor activity, thereby inhibiting tumor growth (Wang et al., 2010; Zhao et al., 2016). NF-κB activator protein in glioma modulates stromal cell-derived factor 1 and macrophage colony-priming factor by targeting Notch1 (Gu et al., 2019). The Notch pathway affects the growth of tumor cells and the function of immune cells in TME, and treatment targeting Notch signaling pathway may be effective. Given that a diversity of immune cells in TME and the regulation of Notch signaling is extremely complex. The role of Notch pathway in different TME and its mechanisms still need to be investigated.

The current study identified two cluster subtypes of BCa based on genes that were associated with Notch pathway using the NMF algorithm. After that, Lasso and Cox regression analysis was performed to construct a 6-gene prognostic risk model. According to our study, this model performed well in predicting survival on BCa patients and correlated with clinical features and immune microenvironment. The risk model was established with DTX3L, CNTN1, ENO1, GATA3, MAGEA1, and SORBS2. Based on the corresponding coefficients, a risk score was calculated. Samples were grouped according to their risk levels. Discrepancies between the survival analyses for different risk subgroups were significant. Additionally, risk score was found to be an independent factor of survival. CIBERSORT confirmed that patients in the high-risk subgroup had higher proportions of M0 macrophages and Mast resting cells, while CD8 T cells, T follicular helper cells, T regulatory cells (Tregs), Monocytes, and dendritic activated cells were upregulated in the low-risk group, suggesting different patterns of infiltration among the subgroups. We also demonstrated that different risk subgroup was correlated with various expression levels of checkpoints. IPS scores indicated that patients with different risks respond differently to immunotherapy and low-risk patients may have a better response for immunotherapy. We finally validated the expression of the six risk signature genes in BCa tissue specimens. The qRT-PCR results demonstrated that expression of CNTN1, ENO1, and MAGEA1 were higher in tumor tissues, whereas the expression of GATA3 was higher in normal tissues.

Dysregulation of Notch signaling plays a crucial role in tumorigenesis, and this signaling pathway is highly dependent on the activity of downstream molecules, and its function varies greatly in different cellular environments (Bigas and Espinosa, 2018). Therefore, the specific mechanisms and functions of this pathway are still required to explore.

In this study, we constructed a 6-gene signature associated with Notch pathway, which was an independent prognostic factor in BCa. This 6-gene signature could be recognized as a prognostic marker to reflect the immunity status of BCa. Our study also has some limitations. The clinical data were obtained from the TCGA and GEO public database, so bias was inevitable during the analysis, and these prognostic genes in this model have yet to be tested *in vitro* and *in vivo*.

In conclusion, this study successfully constructed a Notch pathway-related gene model, which can accurately predict the survival prognosis and immune status of BCa patients. These results may provide a basis for future studies on potential individualized treatments for BCa patients in different risk groups.

## Data Availability

The original contributions presented in the study are included in the article/Supplementary Materials, further inquiries can be directed to the corresponding authors.

## Abbreviations

BCa, Bladder cancer; NMF, Non-negative matrix factorization; TCGA, The Cancer Genome Atlas; GEO, Gene Expression Omnibus; OS, Overall survival; PFS, Progression-free survival; TCGA-BLCA, The Cancer Genome Atlas Urothelial Bladder Carcinoma; LASSO, Least absolute shrinkage and selection operator; ROC, Receiver-operating characteristic; AUC, Areas under curve; TME, Tumor microenvironment; GSEA, Gene set enrichment analysis; ICIs, Immune checkpoint inhibitors; IPS, Immunophenoscore; qRT-PCR, Quantitative real time polymerase chain reaction; EMT, Epithelial-mesenchymal transition; PCA, Principal component analysis; t-SNE, t-distributed stochastic neighbor embedding; HPA, Human protein atlas.
