# Supplementary figures and images for: Discovery of Notch Pathway-Related Genes for Predicting Prognosis and Tumor Microenvironment Status in Bladder Cancer
Source: Front Genet. 2022 Jun 30;13:928778. doi: 10.3389/fgene.2022.928778 (PMC9279929; doi:10.3389/fgene.2022.928778)

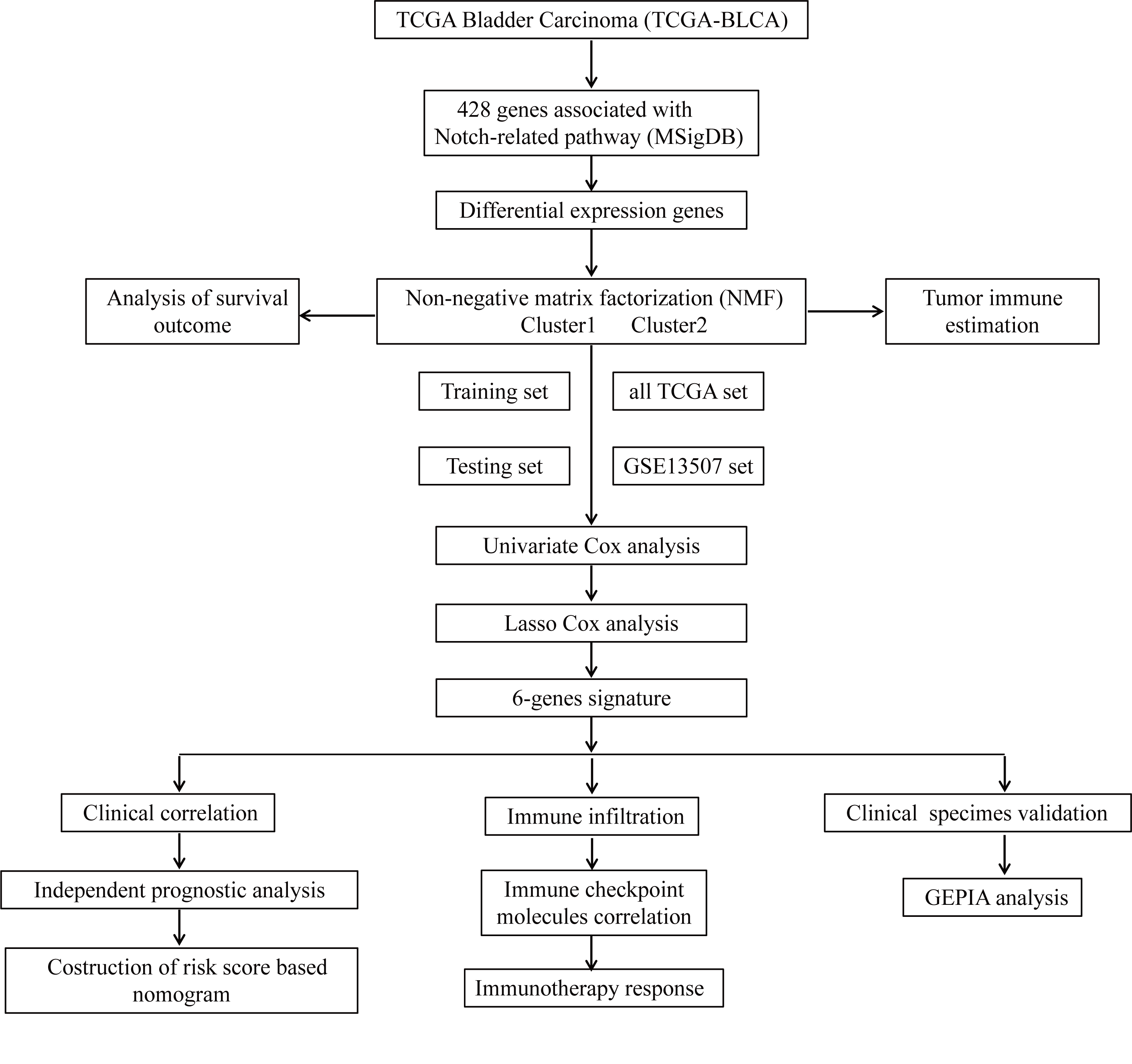

Supplement: Supplementary file 3 [file Image6.TIF]

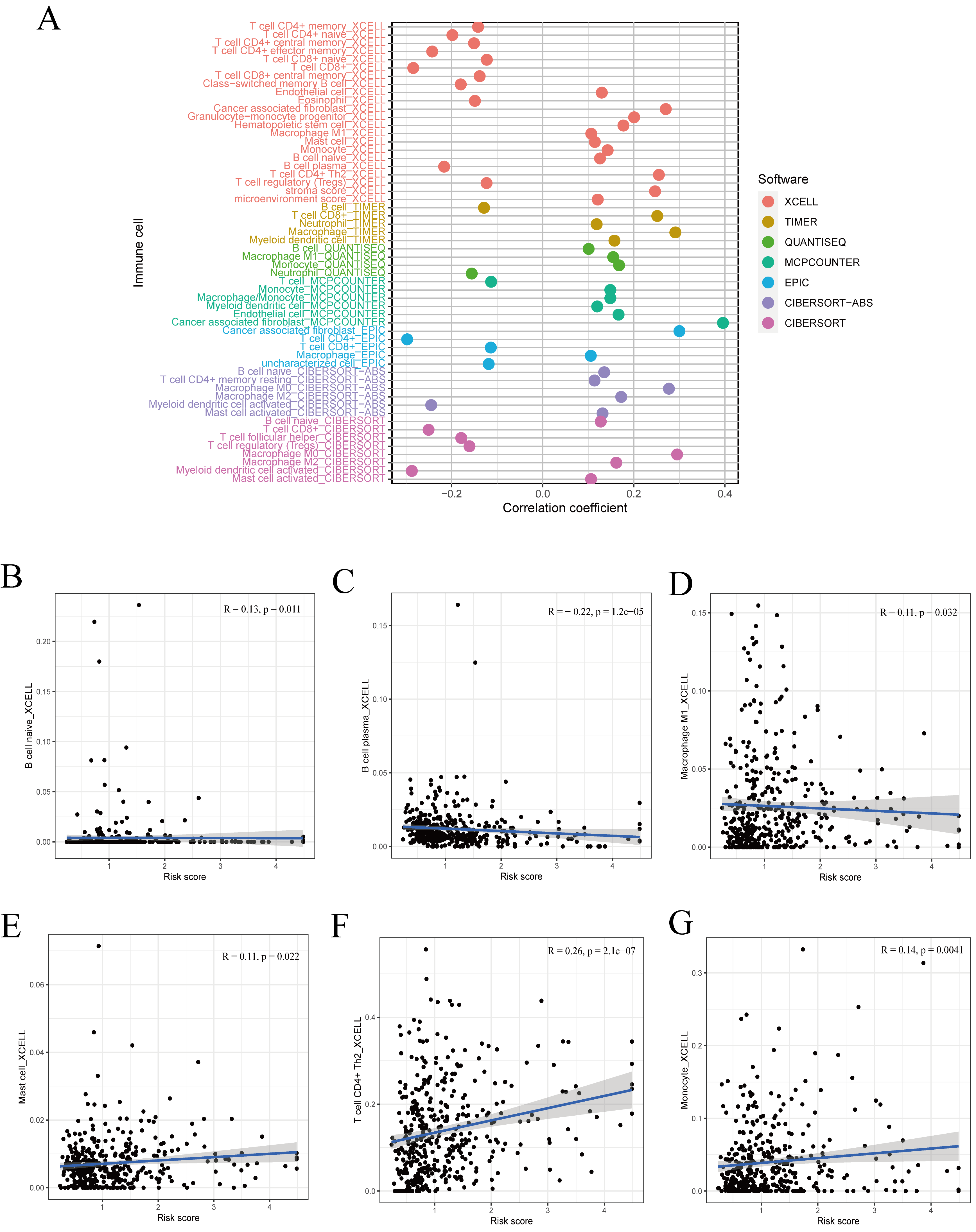

Supplement: Supplementary file 4 [file Image3.TIF]

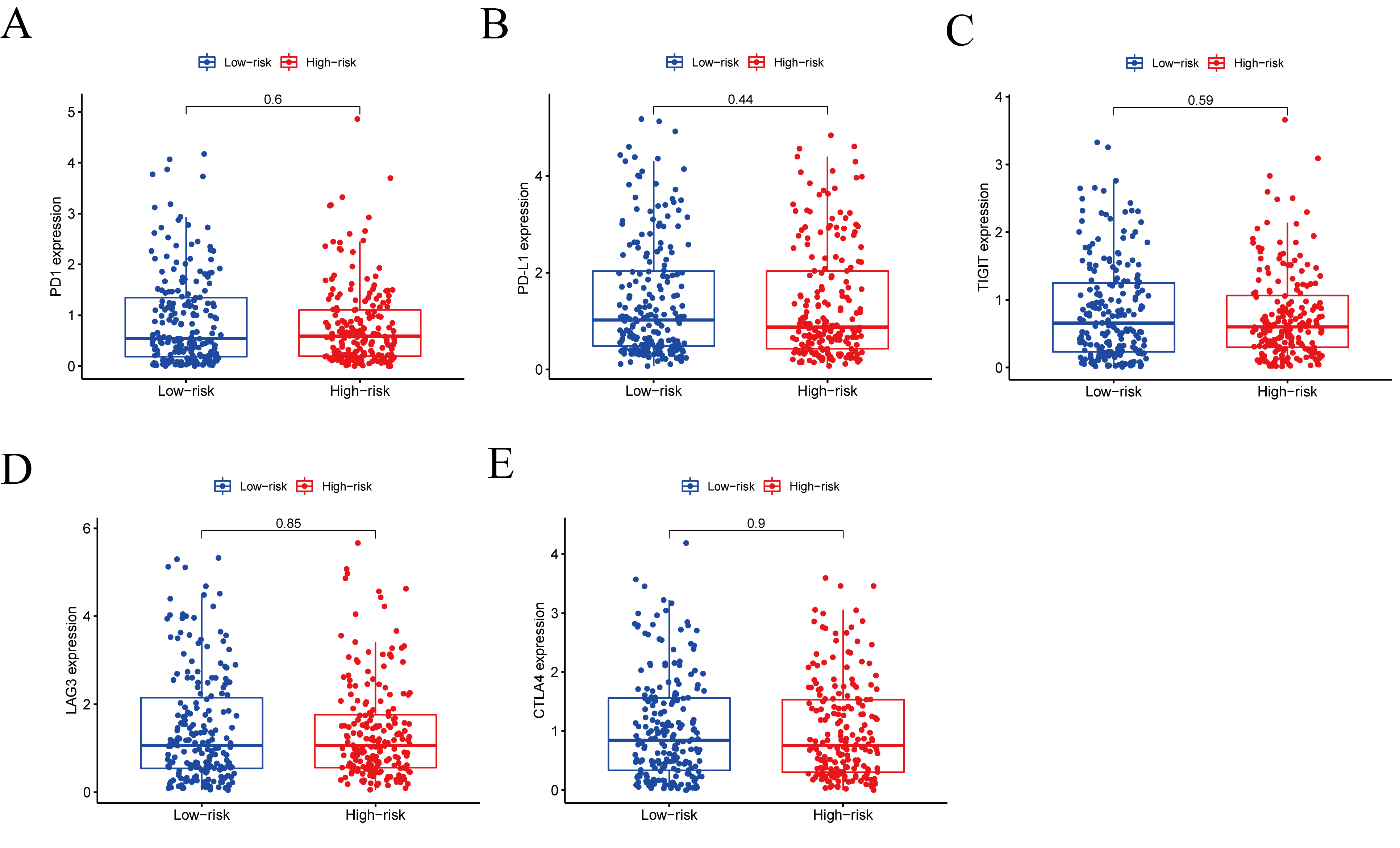

Supplement: Supplementary file 5 [file Image4.TIF]

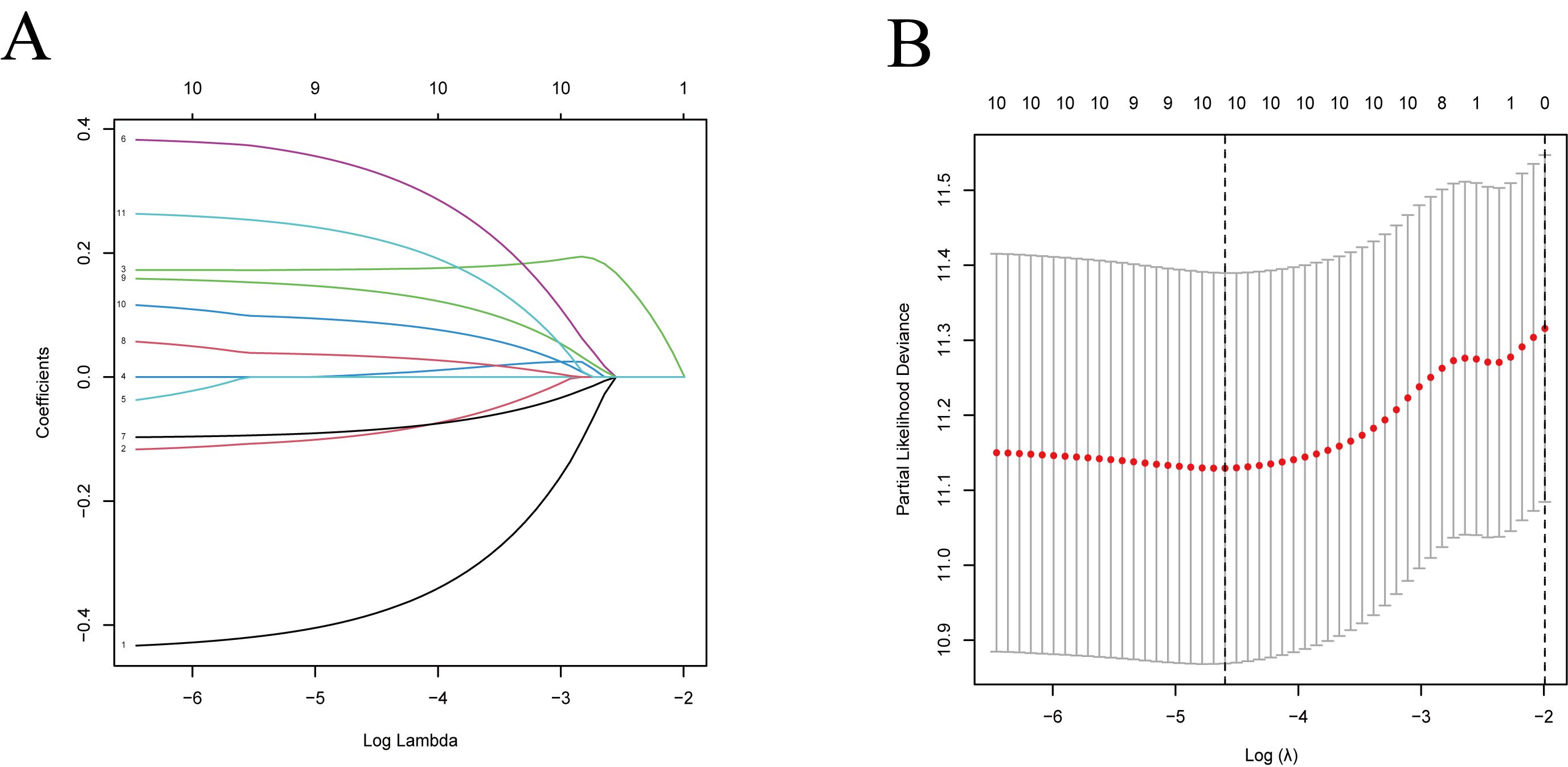

Supplement: Supplementary file 7 [file Image2.TIF]

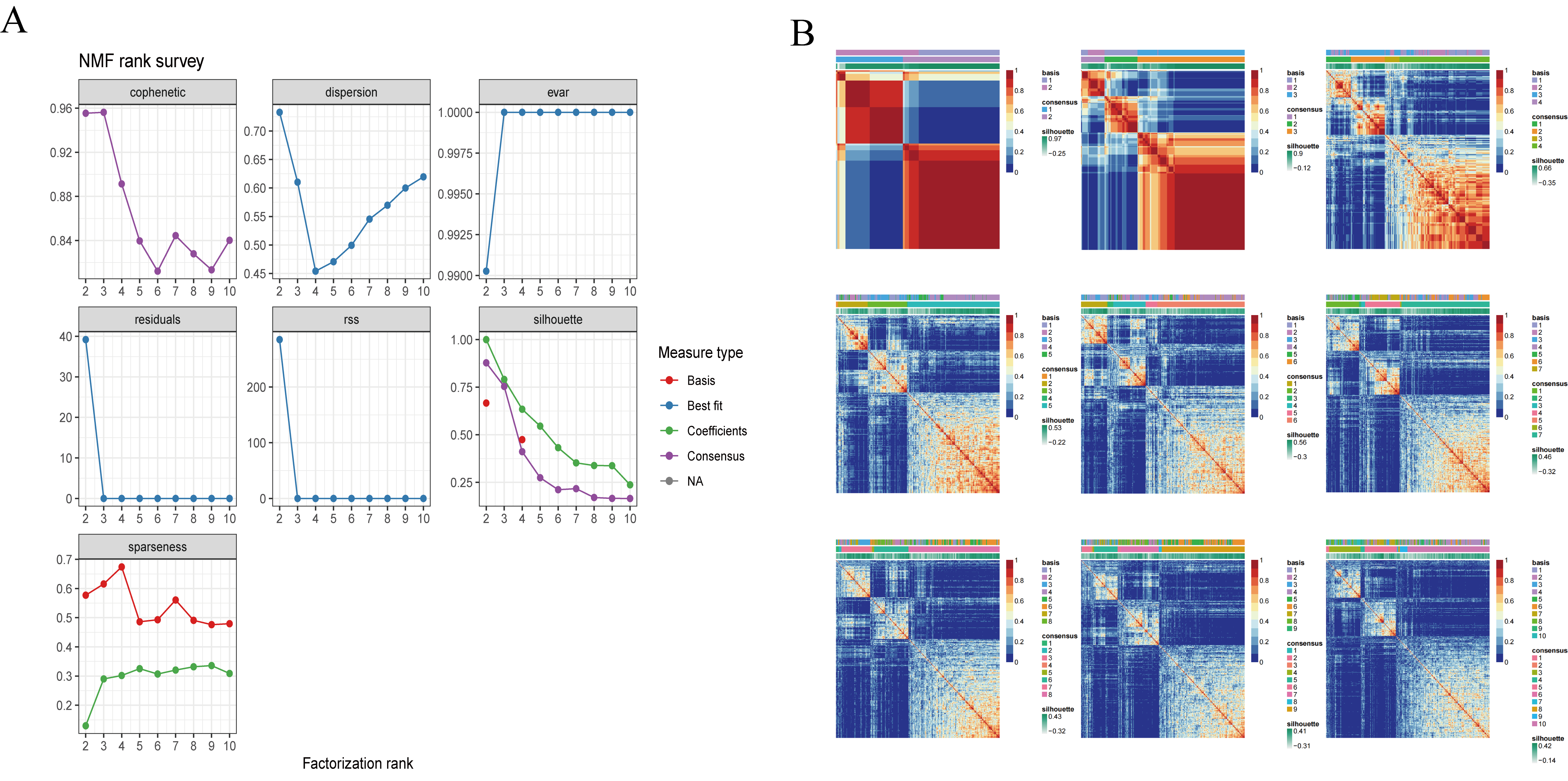

Supplement: Supplementary file 8 [file Image1.TIF]

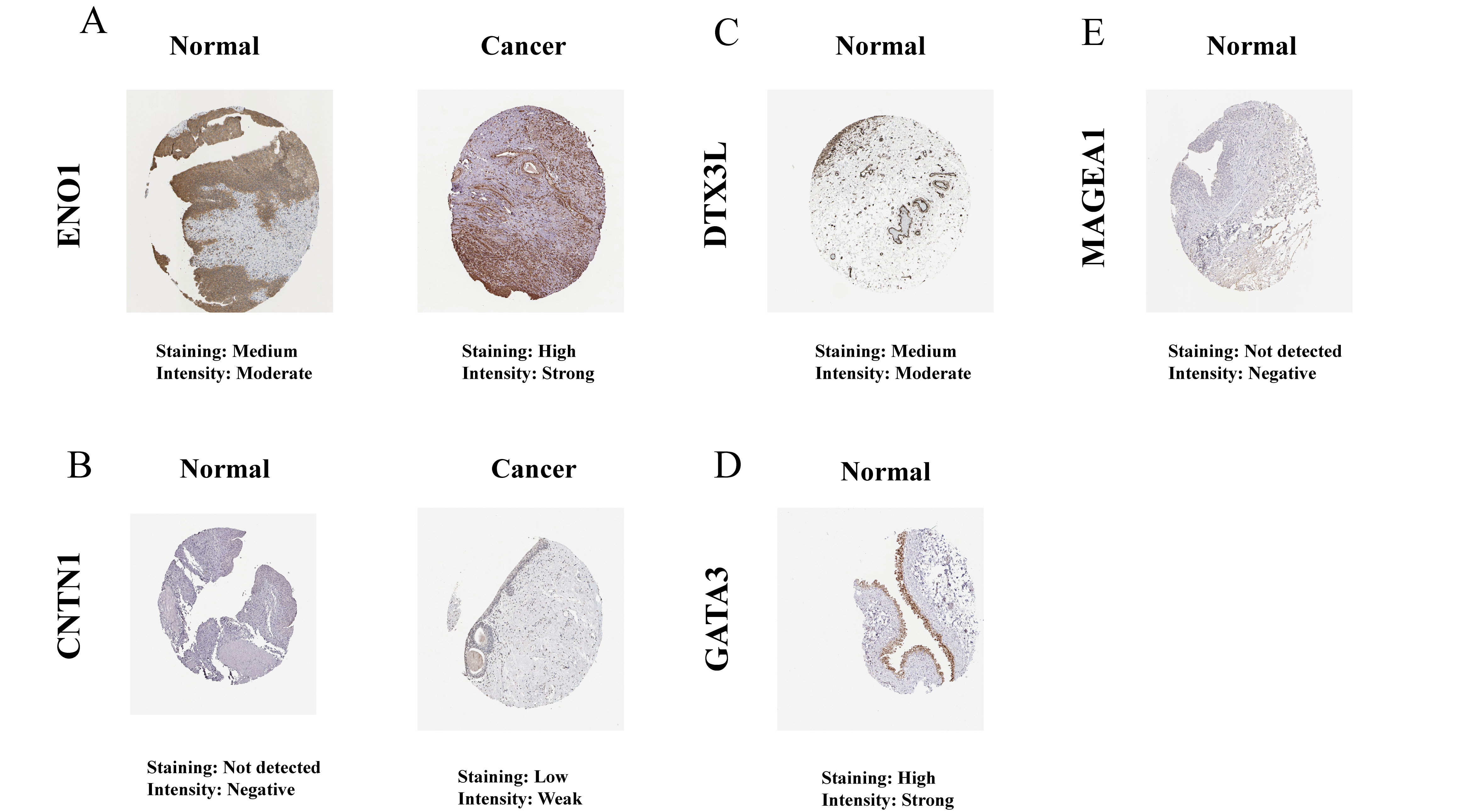

Supplement: Supplementary file 11 [file Image5.TIF]
