# Supplementary material for: Discovery of Notch Pathway-Related Genes for Predicting Prognosis and Tumor Microenvironment Status in Bladder Cancer
Source: Front Genet. 2022 Jun 30;13:928778. doi: 10.3389/fgene.2022.928778 (PMC9279929; doi:10.3389/fgene.2022.928778)
Supplement: Supplementary file 9 [file Table3.DOCX]

**Table S3: Primer information.**

Primer sequence.

| **Gene Name** | **Primer Sequence** |
| --- | --- |
| **β-actin** | F: CACAGAGCCTCGCCTTTGCC  R: ACCCATGCCCACCATCACG |
| **DTX3L** | F: AGTGAAAGGGCAGCTAAGGA  R: GGGCACAGGTTTTTCGTCAA |
| **CNTN1** | F: TTTGCAGCGGAAGTGGGAA  R: TCCTTTGTCTTCCTCAGAAACTCC |
| **ENO1** | F: CTCTTCACCTCAAAAGGTCTCT  R: TCATGGGTCACAGCAGGTTTA |
| **GATA3** | F: TCTGACCGAGCAGGTCGTA  R: TCCTCGGGTCACCTGGGTAG |
| **MAGEA1** | F: CTCTGTGAGGAGGCAAGGTTT  R: GGATCTGTTGACCCAGCAGT |
| **SORBS2** | F: TATCAGGCAGCAGCAAAGGT  R: TCCAATCCAAGTGTCTCTGTCA |

F: Forward; R: Reverse.
