# Supplementary material for: Discovery of Notch Pathway-Related Genes for Predicting Prognosis and Tumor Microenvironment Status in Bladder Cancer
Source: Front Genet. 2022 Jun 30;13:928778. doi: 10.3389/fgene.2022.928778 (PMC9279929; doi:10.3389/fgene.2022.928778)
Supplement: Supplementary file 10 [file Table6.DOCX]

**Table S6. Different clinical outcomes between low- and high-risk group patients among the cohorts.**

***Train cohort***

**Clinical outcome N Risk score  *p***

High risk n(%) Low risk n(%)

Total 283 141 142 0.0003

Live 172 71(50.35%) 101(71.13%)

Dead 111 70(49.65%) 41(28.87%)

***Test cohort***

**Clinical outcome N Risk score  *p***

High risk n(%) Low risk n(%)

Total 120 55 65 0.0661

Live 76 30(54.55%) 46(70.77%)

Dead 44 25(45.45%) 19(29.23%)

***all TCGA cohort***

**Clinical outcome N Risk score  *p***

High risk n(%) Low risk n(%)

Total 403 196 207 <0.0001

Live 248 101(51.53%) 147(71.01%)

Dead 155 95(48.47%) 60(28.99%)

***GSE13507 cohort***

**Clinical outcome N Risk score  *p***

High risk n(%) Low risk n(%)

Total 165 78 87 0.0889

Live 96 40(51.28%) 56(64.37%)

Dead 69 38(48.72%) 31(35.63%)
