# Supplementary material for: Discovery of Notch Pathway-Related Genes for Predicting Prognosis and Tumor Microenvironment Status in Bladder Cancer
Source: Front Genet. 2022 Jun 30;13:928778. doi: 10.3389/fgene.2022.928778 (PMC9279929; doi:10.3389/fgene.2022.928778)
Supplement: Supplementary file 12 [file Table2.DOC]

**Table S2: Seven pathways involved in Notch.**

| **Pathway Database** **Gene Count** |
| --- |
| KEGG_NOTCH_SIGNALING_PATHWAY KEGG 47  REACTOME_PRE_NOTCH_EXPRESSION_AND_PROCESSING Reactome 118  REACTOME_SIGNALING_BY_NOTCH Reactome 245  PID_NOTCH_PATHWAY Pathway Interaction Database 59  HALLMARK_NOTCH_SIGNALING Arthur Liberzon 32  WP_NOTCH_SIGNALING_PATHWAY WikiPathways 63  GOBP_NOTCH_SIGNALING_PATHWAY Gene Ontology 182 |
| Total: 746  Unique: 428 |
